# Contrast-Enhanced Ultrasound of a Hepatic Perivascular Epithelioid Cell Tumor: A Case Report and Literature Review

**DOI:** 10.7759/cureus.81579

**Published:** 2025-04-01

**Authors:** Yun-lei Guo, Ying Dang, Hui Zhang, Jie Lian

**Affiliations:** 1 Department of Ultrasound Medicine, The First Affiliated Hospital of Xi 'an Jiaotong University, Xi‘an, CHN; 2 Department of Medical Imaging, The First Affiliated Hospital of Xi 'an Jiaotong University, Xi‘an, CHN; 3 Department of Pathology, The First Affiliated Hospital of Xi 'an Jiaotong University, Xi‘an, CHN

**Keywords:** borderline neoplasm, ceus, liver tumors, pecoma, rare, ultrasound

## Abstract

Primary hepatic perivascular epithelioid cell tumor (PEComa) is an exceptionally rare mesenchymal neoplasm of borderline malignancy, with limited documentation of its contrast-enhanced ultrasound (CEUS) features in medical literature. In this context, we present a 53-year-old male patient without chronic liver disease, who exhibited a well-circumscribed hypoechoic left hepatic lobe mass on conventional ultrasonography. Subsequent CEUS demonstrated characteristics indicative of malignant tumors, with specific manifestations during the arterial phase, thereby informing clinical decision-making toward surgical resection. Histopathological confirmation of capsular-invasive PEComa underscores the utility of CEUS in characterizing such lesions, particularly in middle-aged patients with incidental solitary hepatic masses lacking predisposing risk factors. These findings advocate for the integration of CEUS into diagnostic algorithms to enhance specificity in atypical hepatic tumor evaluations.

## Introduction

Perivascular epithelioid cell tumors (PEComas) represent a family of mesenchymal neoplasms characterized by histologically distinct perivascular epithelioid cells. While these tumors exhibit multiorgan distribution, they demonstrate a predilection for the retroperitoneum, pelvic cavity, uterus, and gastrointestinal tract. Uterine involvement constitutes the most common presentation, whereas hepatic localization remains exceedingly uncommon [1-5]. Current English-language literature documents fewer than 30 reported cases of primary hepatic PEComas, with contrast-enhanced ultrasound (CEUS) imaging features described in less than 10 instances [6]. 

Hepatic PEComas are tumors of borderline malignant potential, displaying predominantly indolent biological behavior and a female predominance among middle-aged populations. These lesions are frequently clinically silent, accompanied by noncontributory laboratory parameters in most cases [1,2,4]. Conventional ultrasonography remains the cornerstone imaging modality for initial detection, while CEUS offers enhanced diagnostic characterization capability. This study delineates the CEUS features of primary hepatic PEComa and integrates a systematic analysis of existing evidence.

## Case presentation

During hospitalization for management of cardiac insufficiency, a 53-year-old male patient was incidentally found to have a hepatic mass during routine upper abdominal ultrasonographic evaluation at our institution. The patient remained asymptomatic with regard to hepatic pathology, specifically denying constitutional symptoms (fever or chills), gastrointestinal manifestations (abdominal pain, distension, nausea, vomiting, or hematochezia), jaundice, unintentional weight loss, or other disease-related clinical features. Notably, the medical history revealed no documented chronic hepatobiliary disorders or prior oncological diagnoses.

**Figure 1:**
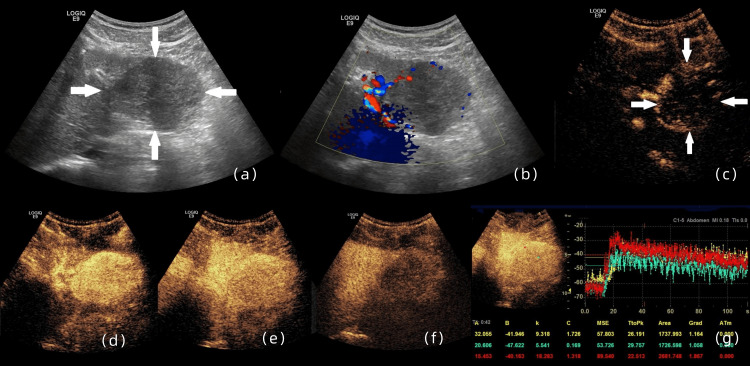
Ultrasonographic characterization of the hepatic lesion (a) B-mode ultrasonography demonstrates a well-demarcated hypoechoic mass (6.5 × 4.8 cm) with ovoid morphology in the left hepatic lobe. (b) Color Doppler imaging identifies circumferential vascularity along the lesion periphery. (c) Early arterial phase (13 seconds postcontrast) exhibits annular hyperenhancement preceding adjacent parenchymal opacification. (d) Complete centripetal filling with homogeneous hyperenhancement is achieved by 19 seconds. (e) Portal phase imaging reveals mild central hypoenhancement, contrasting with sustained peripheral hyperenhancement. (f) Delayed phase (four minutes 25 seconds) demonstrates near-complete contrast washout. (g) Time-intensity curve analysis (ROI annotations: red, perilesional zone; green, central lesion; yellow, adjacent parenchyma) delineates three-phase enhancement kinetics: The enhancement of the lesion preceded that of the adjacent liver parenchyma, with the highest level of enhancement seen at its periphery and the earliest clearance observed in its central part. The area under the time-intensity curve indicated that the lesion periphery had a higher intensity than the adjacent liver parenchyma, which in turn, had a higher level of intensity than the lesional interior

B-mode ultrasonography identified a well-circumscribed hypoechoic mass (6.5 × 4.8 cm) in the left hepatic lobe, demonstrating well-demarcated borders with a near-spherical configuration (Figure 1). The adjacent hepatic parenchyma exhibited homogeneous echotexture without sonographic evidence of fibrotic changes, cirrhotic transformation, or steatotic alterations. Both intrahepatic and extrahepatic biliary systems maintained normal luminal dimensions without ductal dilatation. Color Doppler evaluation revealed circumferential vascularity along the lesion's periphery. Following informed consent acquisition, CEUS was conducted using 2.4 mL of sulfur hexafluoride microbubbles (SonoVue; Bracco Imaging S.p.A., Milan, Italy) administered via the antecubital vein. Contrast dynamics demonstrated initial peripheral enhancement at 12 seconds postinjection (early arterial phase), preceding adjacent parenchymal enhancement onset at 13 seconds, manifesting as an irregular annular hyperenhancement pattern. Rapid centripetal filling progression culminated in complete lesion opacification with homogeneous hyperenhancement by 18 seconds, devoid of peripheral nodular enhancement. Quantitative analysis revealed peak enhancement times of 22.5 seconds (peripheral rim), 29.8 seconds (central zone), and 26.2 seconds (adjacent parenchyma), respectively. Enhancement intensity peaked higher in the peripheral component compared to surrounding liver tissue (reference index 1.0), while central zone peak intensity remained subparendymal (reference index 0.8). Early portal phase imaging (30 seconds) demonstrated initial central contrast clearance with mild hypoenhancement, contrasting with sustained peripheral hyperenhancement. Late portal phase evaluation (90 seconds) revealed progressive peripheral washout initiation. Delayed phase monitoring (four minutes postinjection) documented progressive marked hypoenhancement throughout the lesion. This enhancement pattern evolution comprised three distinct phases: arterial-phase rapid centripetal filling with homogeneous hyperenhancement, portal-phase gradual centrifugal clearance, and delayed-phase complete contrast washout. 

**Figure 2:**
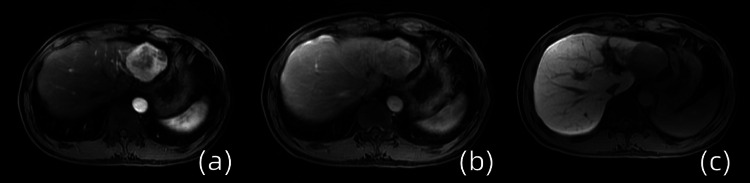
Enhanced magnetic resonance imaging (a) The arterial phase revealed the presence of heterogeneous hyperenhancement. (b) The signal intensity showed a reduction during the venous and transitional phases. (c) The signal intensity decreased during the liver-specific phase

Contrast-enhanced abdominal magnetic resonance imaging (MRI) demonstrated arterial-phase heterogeneous hyperintensity within the lesion, progressing to signal attenuation during portal venous and transitional phases, with eventual hypointensity in the hepatobiliary phase following gadoxetic acid administration (Figure 2). 

Serological analysis revealed elevated alpha-fetoprotein (AFP: 12.6 ng/mL; reference: 0-7.0 ng/mL) and marginally increased carbohydrate antigen 125 (CA-125: 36.2 U/mL; reference: 0-35.0 U/mL), with remaining hematological and biochemical parameters within physiological ranges. 

**Figure 3:**
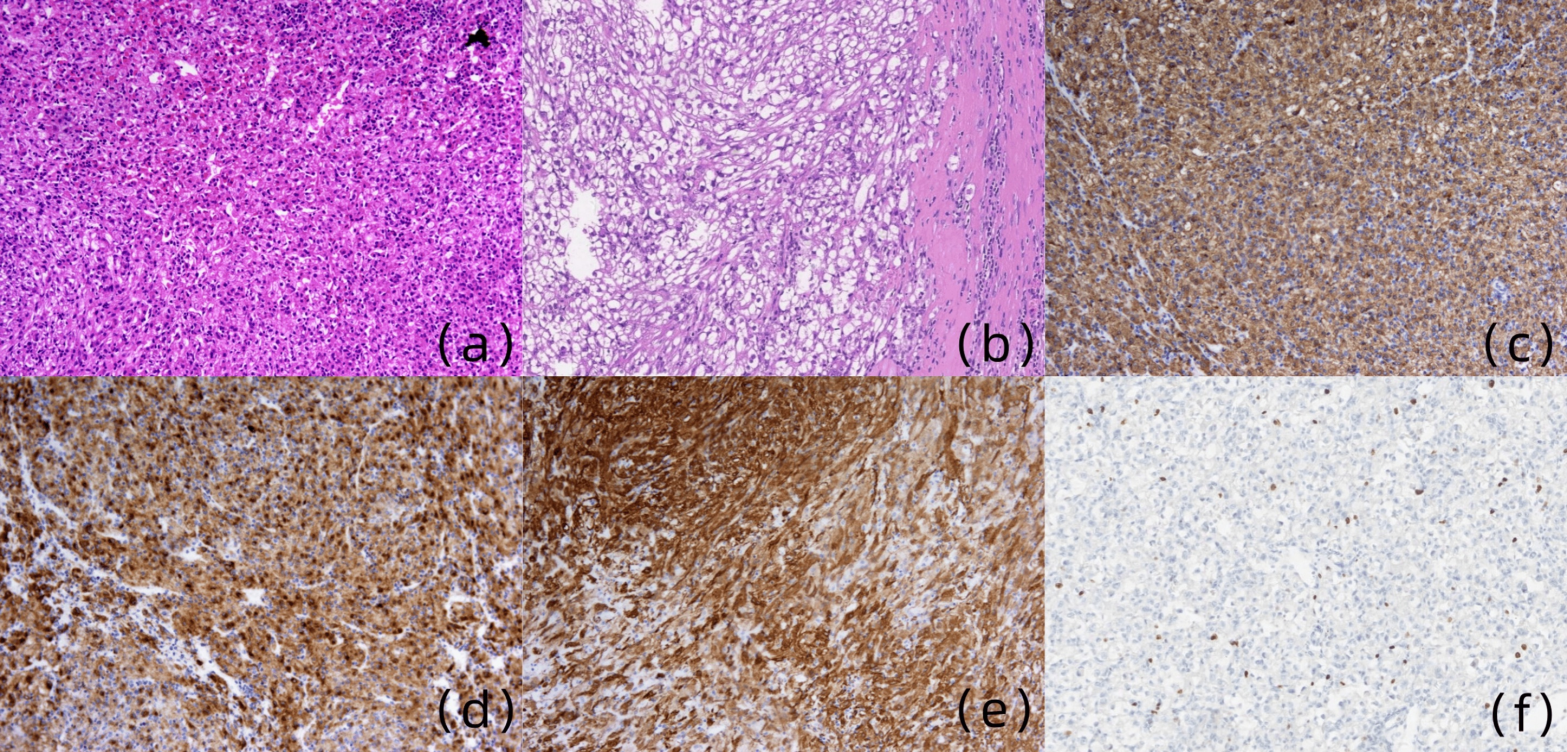
Pathological and immunohistochemical images Pathological and immunohistochemical images. (a) The tumor cells exhibited pleomorphic medium-to-large epithelioid morphology, characterized by abundant cytoplasm and the presence of spindle cell foci surrounding small blood vessels, while lacking adipocytes (haematoxylin & eosin, ×100). (b) The tumor is unencapsulated and invades the adjacent hepatic capsule (hematoxylin-eosin, ×100). (c) The immunohistochemical analysis showed positive HMB45 staining (×100). (d) The immunohistochemical analysis demonstrated positive staining for MelanA (×100). (e) The immunohistochemical analysis showed positive SMA staining (×100). (f) Ki-67 shows low expression.

Surgical resection of the hepatic mass was performed, with histopathological examination confirming a PEComa with capsular infiltration (Figure 3). Immunohistochemical profiling showed strong positivity for melanocytic markers (HMB-45, Melan-A) and smooth muscle actin (SMA) differentiation marker; partial reactivity for mesenchymal markers (vimentin, desmin); and endothelial marker CD34. The Ki-67 proliferative index was quantified at 3%. 

Postoperative surveillance through cross-sectional imaging (ultrasonography or contrast-enhanced CT) at quarterly intervals demonstrated no evidence of tumor recurrence or metastatic dissemination throughout the 12-month surveillance period.

## Discussion

PEComas represent a rare group of mesenchymal neoplasms. The PEComas family of tumors encompasses epithelioid angiomyolipoma, pulmonary clear cell sugar tumor, lymphangioleiomyomatosis, and histomorphologically/immunophenotypically analogous lesions, including primary extrapulmonary sugar tumors, clear cell myolytic tumors, abdominal/pelvic sarcomas with perivascular epithelioid cell differentiation, and soft tissue/visceral PEComas [6]. These tumors demonstrate broad anatomical distribution with marked female predominance (male-to-female ratio: ~1:4), typically presenting between 45 and 50 years of age [1,2,4]. Most lesions remain clinically silent and manifest as painless masses, though nonspecific presentations may occur, including epigastric discomfort, abdominal distension, dyspeptic symptoms, or radiologic suspicion of malignancy [1,4]. Routine laboratory parameters generally remain unremarkable, though serum AFP may show borderline elevation in malignant variants [7]. The observed marginal elevations of AFP and CA-125 biomarkers in this case may suggest malignant potential.

Hepatic PEComas are classified as neoplasms of uncertain malignant potential, with most cases demonstrating benign biological behavior [4]. Nevertheless, the establishment of standardized diagnostic criteria for malignancy risk stratification remains challenging due to diagnostic limitations including case scarcity, histomorphological heterogeneity, inconsistent nomenclature, and organ-specific phenotypic variations [3]. Longitudinal surveillance, therefore, remains critical for definitive biological characterization. Contemporary malignancy stratification models propose five clinicopathological predictors: (1) tumor diameter > 5 cm, (2) infiltrative growth pattern, (3) high nuclear grade/cellularity, (4) mitotic activity exceeding 1/50 HPF, and (5) the presence of coagulative necrosis/vascular invasion [8]. Lesions lacking these features are designated benign, while those demonstrating ≥2 features are classified as malignant; solitary feature presentation indicates intermediate malignant potential. Although most reported hepatic PEComas exhibit benign profiles, documented cases include locally aggressive recurrences and distant metastases. Notably, longitudinal studies reveal malignant progression in some initially benign-diagnosed cases. Amante et al. documented a hepatic PEComa without initial malignant features that metastasized extensively during extended follow-up [9]. This evidence suggests reclassifying all PEComas as lesions with inherent malignant potential, rather than maintaining a distinct benign diagnostic category.

Ultrasonography serves as a frontline imaging modality for hepatic PEComa detection. A longitudinal analysis of clinical and sonographic characteristics was conducted across seven histologically confirmed hepatic PEComa cases (including the index case), with demographic and imaging parameters systematically detailed (Table 1) [1,10-14]. All reviewed lesions were solitary (median diameter: 3.5 cm), with rare reports of multifocal presentations [2]. Sonographically, 57% (4/7) manifested as well-circumscribed hypoechoic masses, though echogenic variability (hyperechoic/isoechoic patterns) has been documented [11,13,15-17]. Color Doppler evaluation demonstrated circumferential vascularity in all lesions, representing a potential diagnostic hallmark [18-20]. CEUS revealed universal arterial-phase hyperenhancement, with one case exhibiting early washout. The present case demonstrated distinctive annular concentric hyperenhancement during the arterial phase, contrasting with the nodular enhancement pattern characteristic of hepatic hemangiomas. Portal-phase imaging showed isoenhancement or mild washout in 86% (6/7) of cases, with hypoenhancement observed in one instance. Delayed-phase analysis documented hypoenhancement in 71% (5/7) of lesions, while two cases maintained mild hyperenhancement. In our patient, portal-phase contrast kinetics demonstrated gradual hypoenhancement progressing to marked washout in late phases, aligning with CEUS features suggestive of hepatic malignancy. Furthermore, the lesion's size exceeding 5 cm, coupled with histopathological evidence of capsular invasion, met established criteria for malignant classification. 

**Table 1:** Clinical and ultrasonography (US) findings reported in literature review Note: Among the seven patients, only two were male. The lesions were solitary, with a median diameter of 3.5 cm and no specific distribution among liver lobes. Most ultrasound findings revealed well-defined hypoechoic nodules, while a few exhibited hyperechoic or isoechoic signals. Color Doppler ultrasound demonstrated peripheral hyperperfusion. Contrast-enhanced arterial phase displayed high enhancement, whereas portal phase and delayed phase showed slightly variable enhancement levels ranging from high to low. Out of the total patient population, 5/7 individuals opted for surgical resection, while 2/7 made this choice upon discovering lesion enlargement during follow-up. Overall prognosis was favorable

Author	Sex/age	Location in the liver	Max. diameter	Conventional ultrasound	Contrast-enhanced ultrasound	Therapy	Progression-free survival
Matrood et al. [1]	F/51	Segment 5	2.0 cm	Well-defined, hypoechoic, peripheral hyperperfusion	Arterial phase: hyperenhancement. Portal phase: isoenhancement. Late phase: central washout	Surgery	>24 months
Panahova et al. [10]	M/38	Segment 5	3.7 cm	Inhomogeneous tumor with hypoechoic rim, peripheral hyperperfusion	Arterial phase: hyperenhancement. Portal phase: isoenhancement. Late phase: central washout	Surgery	>12 months
Akitake et al. [11]	F/36	Segment 2	3.5 cm	Well-defined, isoechoic, peripheral hyperperfusion	Arterial phase: Hyperenhancement and fast washout. Portal and Late phase: hypoenhancement	Surgery	>18 months
Li et al. [12]	F/36	Segment 6	4.2 cm	Well-defined, hypoechoic, peripheral hyperperfusion	Arterial phase: hyperenhancement. Portal phase: isoenhancement. Late phase: hypoenhancement	Surgery	>16 months
Della Vigna et al. [13]	F/46	Segment 3	3.5 cm	Well-defined, hyperechoic, peripheral hyperperfusion	Arterial phase: hyperenhancement. Portal phase: isoenhancement. Late phase: weak hyperenhancement	Imaging follow-up	The lesion grew by 60% in five months
Dežman et al. [14]	F/24	Segment 4	2.0 cm	Well-defined, hypoechoic, peripheral hyperperfusion	Arterial phase: hyperenhancement. Portal and late phase: iso- to hyperenhancement	Imaging follow-up	The lesion had grown to 2.5 cm after six months
The current case	M/53	Segment 2	6.5 cm	Well-defined, hypoechoic, peripheral hyperperfusion	Arterial phase: annular, centripetal hyperenhancement. Portal phase: mild washout. Late phase: hypoenhancement	Surgery	>6months

Accurate differentiation of PEComas from other hepatic lesions with comparable CEUS features, including hepatocellular carcinoma (HCC), metastatic tumors, cavernous hemangiomas (HCH), hepatocellular adenoma (HCA), and focal nodular hyperplasia (FNH), constitutes a critical diagnostic consideration. HCC typically manifests in patients with viral hepatitis or cirrhotic backgrounds, accompanied by elevated AFP levels, intense arterial-phase hyperenhancement, and subsequent portal/equilibrium-phase contrast washout. Distinguishing AFP-elevated PEComas from HCC presents diagnostic challenges. Metastatic lesions generally correlate with confirmed primary malignancies, frequently demonstrating necrotic components and calcifications. These typically exhibit arterial-phase rim enhancement with early portal-phase washout. HCH displays characteristic arterial-phase peripheral nodular enhancement with centripetal progression, followed by gradual contrast fill-in during the portal phase and persistent delayed-phase retention. HCA predominantly affects reproductive-aged women with oral contraceptive use, demonstrating homogeneous arterial enhancement and delayed-phase iso- to hypoenhancement with well-defined margins. Larger HCA lesions demonstrate increased susceptibility to hemorrhagic complications or rupture. FNH is pathognomonically identified by central stellate scarring with arterial-phase eccentric hyperenhancement and sustained enhancement through portal/equilibrium phases [2,4,15,16]. 

Hepatic PEComas typically demonstrate heterogeneous hypoattenuating masses with circumscribed or infiltrative margins on CT, as documented in the literature [8,19,20]. Malignant variants frequently exhibit heterogeneous architecture with prominent necrotic cores or microcalcifications [7]. Contrast-enhanced CT reveals peripheral heterogeneous enhancement during the early arterial phase, occasionally containing nonenhancing components, followed by progressive attenuation in the portal phase and iso- to hypoattenuation in the equilibrium phase [6,10]. MRI characteristics predominantly include T1-hypointense and T2-hyperintense signal characteristics without macroscopic fat content [12]. Postcontrast sequences demonstrate intense arterial-phase hyperenhancement, significant portal-phase attenuation, and late parenchymal-phase hypointensity [12]. Angiographic evaluation identifies hypervascular architecture with intralesional arteriovenous shunting or vascular malformations as a diagnostic hallmark [6,19]. Malignant PEComas display elevated metabolic activity on 18F-FDG PET/CT compared to benign counterparts, which generally exhibit no significant tracer avidity.

The definitive diagnosis of hepatic PEComas is established through histopathological examination. Gross pathological evaluation typically reveals light brown, friable lesions with uniform soft consistency upon sectioning [5]. Histologically, these neoplasms demonstrate predominant medium-to-large epithelioid cellular morphology characterized by abundant eosinophilic cytoplasm, central vesicular nuclei, nuclear pleomorphism, and marked chromatic aberration. The tumor architecture frequently exhibits perivascular spindle cell proliferation in association with small-caliber vessels, while maintaining the absence of adipocytic components [5,8]. Immunohistochemical profiling demonstrates consistent strong positivity for melanocytic markers (HMB-45, Melan-A) and SMA, confirming dual myogenic and melanocytic differentiation, a diagnostic hallmark of PEComas [2]. These immunohistochemical findings correlate precisely with our case observations. Additionally, our specimen demonstrated partial immunoreactivity for mesenchymal markers (vimentin, desmin), positive endothelial marker expression (CD34), and a low proliferative index (Ki-67: 3%).

The optimal management strategy for hepatic PEComas, particularly regarding surveillance protocols, remains poorly defined [10]. Surgical resection represents the primary therapeutic intervention, justified by the unpredictable risk of malignant transformation. Existing case reports indicate that core needle biopsy alone may be insufficient for comprehensive malignancy assessment, as aggressive histological features and mitotic activity are more reliably evaluated in complete surgical specimens. For advanced or metastatic cases, potential therapeutic modalities include radiotherapy, systemic chemotherapy, and molecularly targeted agents; however, current evidence regarding treatment efficacy remains inconsistent, warranting further clinical investigation [8].

## Conclusions

The diagnostic consideration of hepatic PEComa in this middle-aged patient, presenting with an incidental, asymptomatic solitary hepatic mass, was critically supported by the integration of CEUS findings with clinical and laboratory data. The absence of chronic liver disease and negative tumor markers excluded common malignancies such as HCC or metastatic lesions, while the CEUS features provided pivotal discriminative value: (1) the arterial-phase annular enhancement pattern, characterized by peripheral hypervascularity with centrifugal filling, diverged from the homogeneous hyperenhancement typical of HCC or the "spoke-wheel" enhancement seen in focal nodular hyperplasia; (2) the mild portal-phase washout, distinct from the rapid washout of HCC or the persistent enhancement of hemangiomas, suggested intermediate biological aggressiveness consistent with PEComa's borderline malignant potential. CEUS dynamically visualized lesion-specific perfusion patterns in real-time, refining diagnostic differentiation while surpassing conventional ultrasound in microvascular/temporal enhancement characterization, with radiation-free advantages enabling serial monitoring. This case underscores CEUS as an indispensable tool for characterizing indeterminate hepatic masses, particularly in atypical demographics, by bridging the gap between nonspecific B-mode findings and definitive histopathology. Future studies should validate CEUS-based diagnostic algorithms to optimize preoperative risk stratification and reduce unnecessary biopsies in similar scenarios.
